# Mutation screening and association study of *RNASEL* as a prostate cancer susceptibility gene

**DOI:** 10.1038/sj.bjc.6602401

**Published:** 2005-02-15

**Authors:** C Maier, J Haeusler, K Herkommer, Z Vesovic, J Hoegel, W Vogel, T Paiss

**Affiliations:** 1 Abteilung Humangenetik, Universität Ulm, Albert-Einstein-Allee 11, 89081 Ulm, Germany; 2 Urologische Universitätsklinik und Poliklinik, Abteilung für Urologie und Kinderurologie, Universitätsklinikum Ulm, Prittwitzstrasse 43, 89075 Ulm, Germany

**Keywords:** prostate cancer, *RNASEL*, German population

## Abstract

To date, germline mutations have been found in three candidate genes for hereditary prostate cancer: *ELAC2* at 17p11, *RNASEL* at 1q25 and *MSR1* at 8p22. *RNASEL*, encoding the 2′,5′-oligoadenylate-dependant RNase L, seems to have rare mutations in different ethnicities, such as M1I in Afro-Americans, E265X in men of European descent and 471delAAAG in Ashkenazi Jews. In order to evaluate the relevance of *RNASEL* in the German population, we sequenced its open reading frame to determine the spectrum and frequency of germline mutations. The screen included 303 affected men from 136 Caucasian families, of which 45 met the criteria for hereditary prostate cancer. Variants were analysed using a family-based association test, and genotyped in an additional 227 sporadic prostate cancer patients and 207 controls. We identified only two sib pairs (1.4% of our families) cosegregating conspicuous *RNASEL* variants with prostate cancer: the nonsense mutation E265X, and a new amino-acid substitution (R400P) of unknown functional relevance. Both alleles were also found at low frequencies (1.4 and 0.5%, respectively) in controls. No significant association of polymorphisms (I97L, R462Q and D541E) was observed, neither in case–control analyses nor by family-based association tests. In contrast to previous reports, our study does not suggest that common variants (i.e. R462Q) modify disease risk. Our results are not consistent with a high penetrance of deleterious *RNASEL* mutations. Due to the low frequency of germline mutations present in our sample, *RNASEL* does not have a significant impact on prostate cancer susceptibility in the German population.

Familial clustering of prostate cancer, observed in up to 27 percnt; of all cases, has pointed at the existence of major susceptibility genes that confer a high disease risk. To date, three strong candidates are known, *RNASEL* at 1q25 (Carpten *et al*, 2002), *ELAC2* at 17p11 (Tavtigian *et al*, 2001) and *MSR1* at 8p22 (Xu *et al*, 2002), each having been identified with germline mutations in prostate cancer families. Mapping of these genes was preceded by a history of whole genome and candidate region linkage analyses worldwide (for a review, see Schaid, 2004). Since evidence of linkage at particular susceptibility loci seemed to differ substantially between individual populations, a variable prevalence of particular susceptibility genes must be taken into consideration. The aim of the present study is to evaluate the impact of *RNASEL* on prostate cancer risk in the central European population of Germany.

The *RNASEL* gene encodes the 5′–2′-oligoadenylate-dependant RNase L, a mediator of the interferon-induced RNA degradation pathway. Its tumour suppressor potential has been postulated since the introduction of truncated RNase L protein in murine cells abolished the antiproliferative effect of interferon (Hassel *et al*, 1993). The *RNASEL* gene locus at 1q25 (HPC1) has been implicated in prostate cancer susceptibility as a result of the first genome wide linkage scan, which included 91 hereditary prostate cancer pedigrees from North America and Sweden (Smith *et al*, 1996). Linkage to HPC1 seemed to be stronger in families with early-onset disease. Subsequently, in the initial family collection, the nonsense mutation E265X was discovered in a Caucasian pedigree. A second deleterious variation, M1I, was found in a family of African descent (Carpten *et al*, 2002). Follow-up studies revealed a frameshift mutation, 471delAAAG, as a founder allele in Ashkenazi Jews (Rennert *et al*, 2002).

In order to determine the frequency and spectrum of *RNASEL* germline mutations in our population, we sequenced 303 cases representing 136 prostate cancer families from Germany, Central Europe. Conspicuous variants were examined by a family-based association test and were genotyped in an additional 227 sporadic cases and 207 controls. Genotypes were further analysed for a potential correlation with early disease onset.

## PATIENTS, MATERIALS AND METHODS

### Patients

All individuals described in this report are participants in the Prostate Cancer Genetics Project of the University of Ulm. Urologists from all over Germany were asked to inform all patients with prostate cancer about our project and to motivate them to contact our institution. Although we did not apply any selection criteria to the primary sampling of these prostate cancer patients, most of them were contributed by Urological Surgical Departments from Southern Germany and the majority were treated by radical prostatectomy. The index's probands' self-reported family history of prostate cancer was used to identify prostate cancer families. In addition, a detailed family questionnaire was used as a guide for the recruitment of affected and relevant unaffected relatives. In all cases, the diagnosis of prostate cancer was confirmed by a histopathological report or by another suitable medical record. All probands were of Caucasian descent. In approximately one-third of patients, prostate cancer was diagnosed from their symptoms. In two-thirds of patients, the disease was detected by PSA screening. Informed consent, according to the Institutional Review Board at the University of Ulm, was mandatory.

The families used in this study were required to have at least two relatives with confirmed prostate cancer from which blood samples could be made available. The screening included 136 families with confirmed Mendelian inheritance patterns, of which 45 (33%) matched the criteria for hereditary prostate cancer according to Carter *et al* (1993). In all, 58 families (43%) had two affected relatives, 42 (31%) had three, 29 (21%) had four, and seven (5%) had five or more affected relatives. Overall, 303 familial prostate cancer cases were available for genotyping (2.2 per family). In these probands, the mean age at diagnosis was 63.8 years (47–83 years).

Sporadic cases, where a negative family history of prostate cancer was reported, were recruited at the University of Ulm (*n*=227). The mean age of diagnosis was 64.1 years (range: 42–84). For controls we used 207 healthy, elderly men who were not diagnosed with prostate cancer before. In all, 171 (83%) of the controls were confirmed to have a negative DRE finding. PSA levels were available for 72 (35%) controls and were inconspicuous in these subjects. The mean age at the time of sampling was 57.1 years (range 32–88).

### Genetic analysis

DNA was extracted from peripheral blood lymphocytes by standard procedures. The genomic DNA served as template for PCR amplification of the coding region of *RNASEL* with the use of three primer pairs for the large exon 2, and one pair for each of the exons 3–7. Oligonucleotides and PCR conditions are given in Table 1. PCR samples were cleaned up by filtration membrane plates (Millipore, Schwalbach, Germany) prior to sequencing. PCRs destinated for SNP genotyping received enzymatic treatment using 2.5 U shrimp alkaline phosphatase and 1.0 U exonuclease 1 (USB, Cleveland, USA) per 10 ng PCR product. In the case of multiplex genotyping, PCR products were pooled prior to the cleaning-up step.

In order to sequence the entire open reading frame from both directions, sequencing reactions were set up for each PCR amplification primer and each of the four additional primers within exon 2 (Table 1). We used BigDye version 3.1 (Applied Biosystems, Foster City, USA) as recommended by the manufacturer in a total volume of 5 *μ*l. Samples were lastly filtrated and analysed on an ABI3100 instrument. Individual sequences were aligned to a genomic reference sequence (GenBank accession no. AL138776).

SNP genotyping was performed by a ddNTP primer extension method. Five nucleotide variations (nt289, nt793, nt1199, nt1385, nt1623) were genotyped simultaneously in a multiplex assay using oligonucleotides distinguishable by length. Variation site nt407, identified later, was assayed separately. Sequences and individual concentrations of all primers are given in Table 1. On the PCR templates described above, these primers were elongated at the variation sites by fluorescently labelled ddNTPs in total reaction volumes of 5 *μ*l, using the multiplex SNaPshot kit (Applied Biosystems, Foster City, USA). Residual ddNTPs were digested by calf intestine phosphatase (USB, Cleveland, USA) incubation, using 0.5 U of enzyme. Fragments were separated and detected with an ABI3100 sequencing system.

### Statistical analysis

Family-based association tests were performed with the aid of the FBAT (Horvath *et al*, 2001) programme, which is suitable to estimate transmission patterns within various types of pedigree structures. FBAT analysis is used here for each individual marker to test the null hypothesis of no association in the presence of linkage. The test statistic is based on a linear combination of offspring genotypes and traits, called S. Here, phenotypes were specified exclusively for affected persons (trait score 1), while unaffected and unknown persons were included only to contribute genotype information (trait score 0). Genetic variants were analysed under the genetic model of ‘additive’ effects. Under these conditions, *S* reflects the difference between the observed number of transmitted A1 alleles at a biallelic locus (with alleles A1 and A2) to the affected offspring, and the number *E*(*S*) expected under the null hypothesis. *S* is standardised by dividing it by its standard deviation, the square root of its variance Var(*S*), to form a *Z*-value. Since genotypes within pedigrees may be correlated solely due to the presence of linkage (cosegregation), we used the empirical variance option in order to adjust for this correlation. The *Z*-value was finally transferred into a *P*-value.

For case–control analyses, associations between genotypes and disease status were assessed by unconditional logistic regression using the software package StatView (SAS, Cary, USA). In this context, odds ratios, their 95% confindence intervals and the corresponding *P*-values are given. Age of disease onset data from individuals of different genotypes were compared by two-sided *t*-tests.

## RESULTS

### Variants discovered by the sequencing of *RNASEL* in familial prostate cancer probands

**Figure 1:**
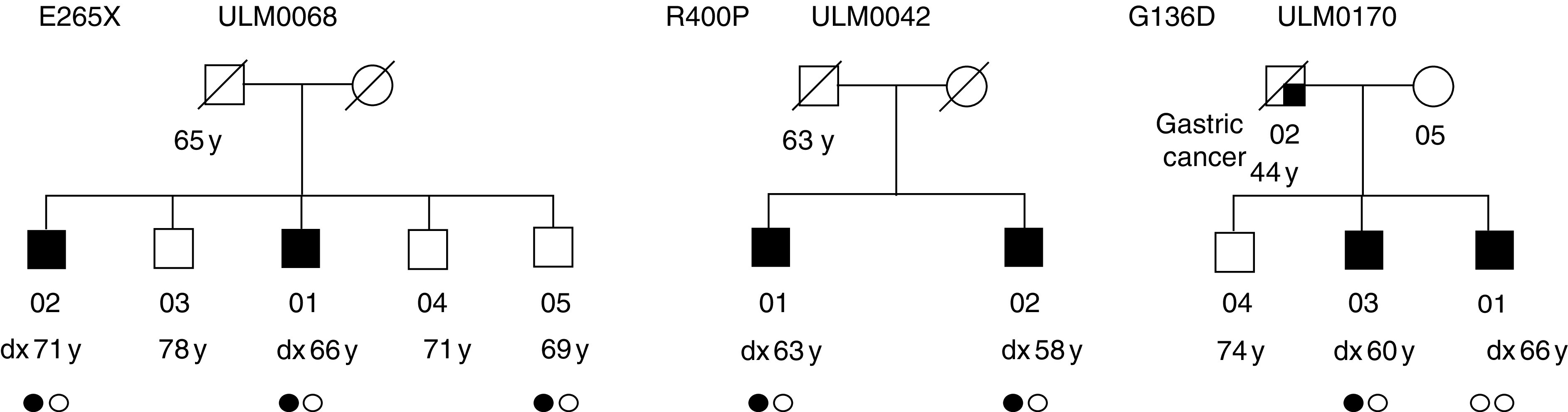
Pedigrees with conspicuous *RNASEL* alleles. Carriers of variants are indicated by one filled circle. Two open circles represent wild-type genotypes.

All sequence variations identified within the open reading frame of *RNASEL* are summarised in Table 2 . Among 303 probands from 136 prostate cancer families, we discovered one protein-truncating variant, nt g793t, that resulted in the previously reported premature stop codon E265X. This point mutation was present in two affected brothers of a single family (Figure 1, family ULM0068). A third brother (individual ULM0068_05), who was available for genotyping, was analysed subsequently. It was discovered that he too carried variant E265X, but was to date (69 years of age) not diagnosed with prostate cancer. In all three siblings, the nonsense mutation was coincident with a further amino-acid change (G49S). According to earlier reports (Carpten *et al*, 2002; Rokman *et al*, 2002), this seems to be part of a unique founder haplotype. A second conspicuous finding was a previously unreported missense variation (R400P). The arginine-to-proline substitution affected the protein kinase-like domain of RNase L; however, its functional relevance remains to be determined. The causal nucleotide exchange, g1199c, cosegregated with the disease in an affected sib pair with no additional relatives available for genotyping (Figure 1, family ULM0042). One further rare amino-acid exchange (G136D) was identified in a single proband, but was not present in his affected brother (Figure 1, family ULM0170). Aside from these rare variants, we also observed the common amino-acid substitutions I97V, R462Q and D541E. Silent exonic variations included g1179a, c1584a and g2172a.

### Family-based association test

A family-based association test was applied in order to examine the transmission of peptide variants to affected individuals. In order to increase the information on parental genotypes, an additional 110 nonaffected male and female family members were tested at the variation sites G136D, E265X, R400P, I97V, R462Q and D541E. The rare variants G136D, E265X and R400P were not recovered in families other than those described above (Figure 1). All the three were not informative for the analysis. The results of the remaining polymorphisms are shown in Table 3. A tendency of overtransmission was observed for the infrequent valine variant at position 97 (*P*=0.17), although only two families contributed to the test. In contrast, sufficient numbers of informative families (39 and 34, respectively) were available for the common variants R462Q and D541E. The observed transmissions towards affected offspring were approximately equal to the expected values, that is, 76 *vs* 76.2 (*P*=0.96), in case of the Q allele at 462, and 81 *vs* 77.4 (*P*=0.37) for the E allele at 541.

### Case–control study of *RNASEL* peptide variants

The genotype frequencies of G136D, E265X, R400P, I97V, R462Q and D541E were determined in 227 prostate cancer patients with no familial disease history (sporadic cases) and in 207 healthy men (controls). Prior to case–control analyses, the observed genotype counts at all loci were verified to resemble the Hardy–Weinberg equilibrium: in the control group (*P*>0.51), the sporadic group (*P*>0.45) and in the group of index probands from prostate cancer families (*P*>0.64). The exchange G136D was seen neither in sporadic cases, nor in controls. The observations at all other variation sites are given in Table 4. Overall, we found that none of the genotypes were significantly associated with either familial, sporadic or the combined group of prostate cancer. Variants that tended to be over-represented in cases include the newly identified missense exchange R400P, which was recovered in two (0.9%) sporadic prostate cancer cases and in one (0.5%) nonaffected man (OR=1.83; CI95=0.17–20.3; *P*=0.62). Also, heterozygous carriers of the valine allele at codon 97 seemed to be increased in sporadic cases (1.8 *vs* 1.0% OR=1.84; CI95=0.33–10.1, *P*=0.49) and were most frequent in familial probands (3.0% OR=3.13; CI95=0.57–17.3; *P*=0.19). Rather similar genotype frequencies in the study samples were found for the common polymorphisms R462Q and D541E. An inverse distribution was observed for the nonsense mutation E265X, which was found twice in prostate cancer cases (0.6%, one familial and one sporadic proband) and three times (1.5%) in controls (OR=0.38; CI95=0.06–2.27; *P*=0.29).

### Effect of *RNASEL* variants on early-onset prostate cancer

All 530 prostate cancer patients genotyped for this study were analysed for a correlation of genotype and age of disease onset. In a subgroup of 49 cases who were diagnosed at the age of 55 years or younger, which is commonly assigned as early-onset prostate cancer, none of the genotypes were significantly over-represented (Table 5). However, regarding the mean age of onset, the four carriers of the R400P exchange appeared to be 2 years younger than wild type (*t*-test; *P*=0.24).

## DISCUSSION

The *RNASEL* gene at 1q25/HPC1 is one of three strong candidate genes for hereditary prostate cancer known to date. Since deleterious germline mutations were identified in *RNASEL*, growing evidence has been reported for the causal quality of the potential tumour suppressor gene. Firstly, prostate carcinomas of mutation carriers were shown to exhibit loss of heterozygosity (LOH), and, as a consequence, were deficient in functional RNase L (Carpten *et al*, 2002). Secondly, a common amino-acid variant, R462Q, which was previously found to be associated with prostate cancer risk (Casey *et al*, 2002), was proven to reduce the affinity of RNase L to form catalytically active dimers (Xiang *et al*, 2003). However, the evaluation of the gene's impact on prostate cancer susceptibility requires a sufficient series of epidemiologic data. Crucial issues include the estimation of the disease risk for mutation carriers and the description of allele frequencies, which may differ between populations due to the given locus heterogeneity of prostate cancer.

In the present screening of 136 German prostate cancer families, we discovered two novel missense mutations, aside from the nonsense mutation E265X. While one of these amino-acid exchanges (G136D) seemed to be a private mutation in one single proband, the variant R400P appeared to be present in the population with a low allele frequency. There are some indications towards an effect of R400P, like cosegregation with the disease in the affected sibpair, a higher frequency of carriers among prostate cancer cases, and a younger age of these patients. An association with the disease, however, remains unknown, due to the lack of statistical significance.

On the other hand, no remarkable proportion of our families could be explained by *RNASEL* mutations like E265X, although presently we cannot fully rule out gene deletions or epigenetic changes which are undetectable by the procedure of exon sequencing. Consistent with a low number of deleterious *RNASEL* variants in our study sample was our recent genomewide linkage scan, resulting in an insignificant *Z*_LR_ score of 1.28 (*P*=0.10) at marker D1S218 at 1q25/HPC1 (Maier *et al*, 2004). However, in contrast to the entire family collection, some individual pedigrees have supported linkage to HPC1. The family with the highest individual evidence (*Z*_LR_=2.86, *P*=0.002), including four affected first-degree relatives, was checked for *RNASEL* expression in order to find further clues for hidden deleterious mutations. In every proband, transcripts of both alleles were confirmed to be present at equal amounts, and to be spliced correctly (data not shown). Taken together, these results indicate that *RNASEL* could only account for a minor portion of familial prostate cancer in Germany. Similar conclusions have been drawn from other Caucasian populations, where, overall, E265X has so far been observed as the only truncating *RNASEL* mutation. It appeared together with the rare missense variant G49S in study samples from North America, Scandinavia, and also in Germany, indicating a founder haplotype of European descent (Carpten *et al*, 2002; Rokman *et al*, 2002, Chen *et al*, 2003). However, an appreciable carrier frequency of 4.3% was identified only in families from Finland, with some lack of cosegregation with the disease (Rokman *et al*, 2002). Similar to our study sample, a combined family collection from the United States and Sweden revealed rare carriers of E265X (Chen *et al*, 2003), while no mutations at all were found in a further American study (Wang *et al*, 2002).

It is noteworthy that we observed a higher frequency of E265X mutation carriers in healthy men than in prostate cancer patients. This rather unexpected finding could be consigned to chance, owing to our limited sample size. Additional genotyping of 190 unselected blood donors revealed no further heterozygotes of E265X, suggesting that the frequency in our population could indeed be somewhat lower. A crucial issue is the validity of the disease-free status of the three controls carrying the truncating variant. All the three had a negative DRE, and PSA values were recorded for two of the men, which were 0.1 ng ml^−1^ by the age of 64 and 0.5 ng ml^−1^ by the age of 55. The prevalence of prostate cancer is as low as 6.6% among men with these PSA levels (Carter, 2004). Additionally, in our single E265X-positive prostate cancer family, we identified a male carrier with no evidence of the disease (DRE normal; PSA=1.7 ng ml^−1^) at 69 years of age. These data do not support a high penetrance of deleterious *RNASEL* mutations.

Regarding the common variants of *RNASEL*, we did not observe any significant association, neither with prostate cancer in general nor with early-onset disease. We cannot exclude that our sample size may be too small to sufficiently assess a moderate disease effect of low frequent variants, such as the amino-acid exchange I97L, which tends to be over-represented in sporadic and familial prostate cancer. On the other hand, our results regarding frequent polymorphisms are valid. Especially, the polymorphism R462Q showed absolutely no correlation with prostate cancer risk in our case–control study. This finding was further confirmed by our negative result of a family-based association test. In contrast to these data, the glutamine residue at 462 was recently suggested to be responsible for up to 13% of prostate cancer cases (Casey *et al*, 2002), and was proven to alter the enzyme's activity (Xiang *et al*, 2003). Despite this impressive result, two further studies found disease risk associated with the more frequent arginine allele (Wang *et al*, 2002; Nakazato *et al*, 2003). These inconsistencies might suggest a *cis*-acting locus in linkage disequilibrium to the R462Q polymorphism, where causal variants may reside on diverse haplotypes and disproportionally show up in different study populations. A similar hypothesis was discussed earlier as a result of Wang *et al* (2002) finding association of the arginine allele exclusively in familial prostate cancer and not in sporadic disease. Such findings argue against the plausibilty of a particular variant always effecting the same functional consequence, unless the true disease effect is driven by an unknown factor in *cis*, which is coincident in a subgroup of alleles. Additional investigations will be needed in order to further characterise the role of R462Q in influencing prostate cancer risk.

## CONCLUSION

Apart from the caucasian founder mutation E265X, a missense change of unknown relevance, R400P, was observed in our study samples. However, *RNASEL* does not account for a significant number of familial prostate cancers in Germany, and the penetrance of deleterious mutations may need further evaluation. Common variants such as R462Q do not seem to influence prostate cancer risk in our study population.

## Figures and Tables

**Table 1:** Oligonucleotides used for PCR amplification, sequencing and genotyping by ddNTP primer extension

**PCR and sequencing primers**		**Annealing temperature (°C)**	**Conc. MgCl_2_ (mM)**

ex2-f1: actgctgctctgttgcca	ex2-r2: agagcatggatcaaggcat	58	1.5
	ex2-1r: cattgacatctgctcctttag	Sequencing only	
Ex2-2h: gtgctgaccctgttctga		Sequencing only	
Ex2-f3: acacgtagaggtcttgaag	ex2-r3: cacaatctgtactggctcc	51	1.5
Ex2-f4: ctggagcaagagcacata	ex2-r5: aatcatccacatttactctag	58	1.5
	ex2-4r: ctcccatagaatgtcacca	Sequencing only	
Ex2-5h: ctacctggggttctatgag		Sequencing only	
Ex3-f: gcagatacctaacagatcaaa	ex3-r: cctactagttctgtccctc	51	1.5
Ex4-h: ctaatagcgtgcaccactc	ex4-r: tgatttgcagcccagactt	60	2.0
Ex5-h: acaccaaaatctaacggtt	ex5-r: cacctctttgagcctct	53	1.5
Ex6-h: gacacaattatagttagcatt	ex6-r: gtaaggcaacagtgatag	53	1.5
Ex7-h: gccatatgctgtgaag	ex7-r: ccaaggactctacagctaa	58	1.5


**ddNTP-extension primers**	**Final primer Conc. (mM)**
	
rl-407: tttttttttt tttttatgga agccgctgtg tatg	0.02
rl-289: ttttttttttgaatggggccacgcctttt	0.12
rl-793: ctggagcaagagcacata	0.03
rl-1199: ttttttttttttttttttttttgagggcagcccac	0.12
rl-1385: tttttttttttttttttttttgaggaagatgaatttgccc	0.08
rl-1623: tttttttttttttttttttttttttcattactttgagctttcag	0.30

**Table 2:** Sequence variants found in 303 familial prostate cancer patients

			**Observed alleles**		
**Location**	**Nucleotide exchange**	**Amino-acid exchange**	**No.**	**Frequency**	**Also found in previous study population**
Exon 2	g175a	G59S	2/594	0.003	USA^a^,^b^, Finland^c^
Exon 2	a289c	I97L	9/600	0.015	USA^a^,^b^,^d^
Exon 2	g407a	G136D	1/600	0.002	Novel
Exon 2	g793t	E265X	2/606	0.003	USA^a^,^b^, Finland^c^
Exon 2	g1179a	—	5/604	0.008	Finland^c^
Exon 2	g1199c	R400P	2/604	0.003	Novel
Exon 2	g1385a	R462Q	245/604	0.406	USA^a^,^b^,^d^, Finland^c^, Japan^e^
Exon 4	c1584a	—	1/606	0.002	Novel
Exon 4	t1623g	D541E	359/606	0.592	USA^a^,^b^,^d^, Finland^c^, Japan^e^
Exon 7	g2172a	—	24/596	0.040	USA^a^,^b^, Finland^c^

a Carpten *et al* (2002)

b Chen *et al* (2003)

c Rokman *et al* (2002)

d Wang *et al* (2002)

e Nakazato *et al* (2003)

**Table 3:** Family-based association tests

**Variant**	**No. of informative nuclear families**	**Observed transmissions *S***	**Expected transmissions *E*(*S*)**	**Variance Var(*S*)**	***Z***	***P***
I97L	2	3	1.9	0.6	1.37	0.17
R462Q	39	76	76.2	16.8	−0.05	0.96
D541E	34	81	77.4	16.2	0.89	0.37

**Table 4:** Frequencies of *RNASEL* protein variants in familial prostate cancer, sporadic prostate cancer and in controls

**Genotype**	**Controls total 207 *n* (%)**	**Sporadic cases total 227 *n* (%)**	**Familial index cases total 136^a^ *n* (%)**	**Sporadic cases *vs* controls OR (95% CI)**	**Familial cases *vs* controls OR (95% CI)**	**All cases *vs* controls OR (95% CI)**
97 IL^b^	2 (1.0)	4 (1.8)	4 (3.0)	1.84 (0.33–10.1)	3.13 (0.57–17.3)	2.32 (0.49–11.0)
265 EX^b^	3 (1.5)	1 (0.4)	1 (0.7)	0.30 (0.03–2.92)	0.50 (0.05–4.89)	0.38 (0.06–2.27)
400 RP^b^	1 (0.5)	2 (0.9)	1 (0.7)	1.83 (0.17–20.3)	1.53 (0.10–24.6)	1.72 (0.18–16.6)
462 RQ	97 (46.9)	102 (44.9)	69 (50.7)	0.86 (0.60–1.31)	1.18 (0.73–1.92)	0.97 (0.66–1.41)
QQ	37 (17.8)	36 (15.9)	23 (16.9)	0.81 (0.46–1.39)	1.03 (0.54–1.96)	0.88 (0.53–1.44)
541 DE	97 (46.9)	112 (49.3)	64 (47.1)	1.13 (0.68–1.88)	1.35 (0.73–2.52)	1.20 (0.75–1.91)
EE	69 (33.3)	73 (32.2)	52 (38.2)	1.03 (0.60–1.78)	1.55 (0.81–2.94)	1.20 (0.73–1.96)

a The proband with whom initial family contact was made (index case) was chosen to represent the family.

b No homozygous genotypes were observed for these rare variants.

**Table 5:** Association of genotypes and age of onset

**Genotype**	***n* a**	**Age mean (s.d.)**	***P*-value^b^**	**Genotypes in early onset^c^** **n (%)**	**Early-onset disease *vs* controls OR (95% CI)**
97 II	514	63.9 (6.6)			
IL	13	65.1 (5.4)	0.44	1 (2.0)	2.14 (0.19–24.0)
265EE	527	63.9 (6.6)			
EX	3	65.7 (4.5)	0.57	0 (0.0)	—
400RR	525	63.9 (6.6)			
RP	4	61.8 (3.0)	0.24	0 (0.0)	—
462RR	193	64.0 (7.1)			
RQ	253	63.7 (6.4)	0.73	21 (42.9)	0.75 (0.38–1.48)
QQ	83	64.3 (5.9)	0.66	7 (14.3)	0.66 (0.26–1.69)
541DD	91	64.6 (7.4)			
DE	261	63.3 (6.4)	0.16	27 (55.1)	1.43 (0.60–3.40)
EE	178	64.4 (6.3)	0.84	14 (28.6)	1.04 (0.40–2.69)

a All genotyped cases, including 227 sporadic cases and 303 familial cases, were analysed.

b *P*-values refer to comparisons of mean age between patients homozygous for the wild-type allele on the one hand, and patients with the altered genotype on the other hand.

c The early-onset subsample comprises 49 probands (28 familial and 21 sporadic cases) who were diagnosed at 55 years of age or younger.
